# Internet-Delivered Cognitive Behavioral Treatment for Chronic Pain in Adolescent Survivors of Childhood Cancer: Protocol for a Single-Group Feasibility Trial

**DOI:** 10.2196/45804

**Published:** 2023-08-01

**Authors:** Michaela Patton, Linda E Carlson, Melanie Noel, Tonya Palermo, Victoria Forster, Sara Cho, Fiona Schulte

**Affiliations:** 1 Department of Psychology University of Calgary Calgary, AB Canada; 2 Seattle Children's Research Institute Seattle, WA United States; 3 The Hospital for Sick Children Toronto, ON Canada

**Keywords:** pediatric oncology, chronic pain, cognitive behavioral therapy, eHealth, intervention, late effects, cancer survivorship, pediatric cancer, youth, mental health, psychological difficulties, chronic health conditions, pain management, intervention, cancer survivor

## Abstract

**Background:**

There are over 500,000 survivors of childhood cancer in North America alone. One in 4 survivors experiences chronic pain after treatment has been completed. Youths with chronic pain report increased anxiety, depression, activity limitations, and sleep disturbances. An 8-week web-based cognitive behavioral treatment for chronic pain (Web-Based Management of Adolescent Pain [WebMAP]) has demonstrated a reduction in pain in youths but has not yet been explored in survivors.

**Objective:**

The objectives of this study are to (1) test the feasibility and acceptability of WebMAP for a sample of survivors with chronic pain and their parents; (2) assess the acceptability of WebMAP using qualitative interviews; (3) assess WebMAP’s effect on activity limitations, pain intensity, depression and anxiety symptoms, and sleep disturbances; and (4) assess WebMAP’s effect on parent pain catastrophizing and parental response to their child’s pain.

**Methods:**

A single-arm mixed methods pre-post intervention study design will be used. Participants will be 34 survivors and at least one of their parents or caregivers. Inclusion criteria are (1) a cancer history, (2) current age of 10-17 years, (3) >2 years post treatment or >5 years post diagnosis, (4) pain present over prior 3 months impairing >1 area of daily life and occurring >1 time per month, and (5) computer access with broadband internet. Survivors will complete a pretreatment questionnaire, which will include the following: the Child Activity Limitations Interview, the pain intensity Numerical Rating Scale, Patient-Reported Outcomes Measurement Information System (PROMIS)–Pain Interference, Anxiety, Depression, Insomnia Severity Index, and Adolescent Sleep Wake Scale. Parents will complete the Pain Catastrophizing Scale–Parent Version and the Adult Responses to Child Symptoms. Upon completion of pretreatment questionnaires (T0), survivors will begin WebMAP. After the 8-week intervention, survivors will complete the same measures (T1), and at 3-month follow-up (T2). Posttreatment interviews will be conducted to determine acceptability. Feasibility will be assessed via recruitment and retention rates. Treatment engagement will be measured by number of modules completed. Pre-post outcome data will be assessed using linear mixed models. Qualitative data will be analyzed using thematic analysis. Patient partners will be involved in study design, recruitment, interpretation of results, and knowledge translation.

**Results:**

This study has been funded in January 2022. Data collection started in May 2022 and is projected to end in August 2023. We have enrolled 10 participants as of December 2022.

**Conclusions:**

Investigating whether WebMAP is useful to survivors will be an important step in improving pain management in this population.

**Trial Registration:**

ClinicalTrials.gov NCT05241717; https://clinicaltrials.gov/ct2/show/NCT05241717

**International Registered Report Identifier (IRRID):**

DERR1-10.2196/45804

## Introduction

Currently, there are over 500,000 survivors of pediatric cancer in North America alone [1]. Unfortunately, two-thirds of survivors of childhood cancer will experience late- and long-term effects from their cancer and its treatments, such as subsequent chronic health conditions and psychological difficulties [2]. Our previous work demonstrated that approximately 1 in 4 survivors of childhood cancer experiences chronic pain after treatment completion and 1 in 5 experiences moderate to severe chronic pain problems, reporting high pain intensity and interference in activities [3]. We also found survivors living with chronic pain reported significantly worse depressive and anxiety symptoms and poorer quality of life than survivors without chronic pain [3,4].

Survivors of childhood cancer are already at risk for worse mental health than their healthy peers as a result of cancer and its often toxic treatments [2], and pain could exacerbate these outcomes. Importantly, pain among survivors of childhood cancer has been shown to persist into adulthood, which among noncancer adolescents has been linked to lower educational and social attainment and poor vocational functioning in young adulthood [5]. With the growing number of survivors of childhood cancer, improving long-term follow-up care will help improve health care for these vulnerable youths by reducing their risk of continued pain and poor health into adulthood.

Chronic noncancer pain in childhood is associated with negative developmental outcomes in young adulthood including a higher likelihood of dropping out of high school, a lower likelihood of graduating from university, poor vocational functioning, and social impairments [6]. A large, longitudinal study demonstrated that chronic pain in adolescence conferred risk for lifetime anxiety and depressive disorders [6].

According to the United Nations Office on Drugs and Crime’s most recent World Drug Report, approximately 585,000 people experienced drug-related deaths and the majority of these deaths could be attributed to opioids [7]. Importantly, there is little evidence for the efficacy of prescription opioids for the treatment of chronic pain, and long-term opioid use for the treatment of chronic pain can cause adverse effects such as overdose, cardiovascular events, and motor vehicle accidents [8,9]. Chronic pain is also a significant financial burden on families, society, and the health care system, costing up to CAD $60 billion (US $46.99 billion) per year in Canada [10], and is one of the most common reasons for seeking health care [11,12]. According to a recently proposed developmental model of chronic pain, strategies for pain prevention and management should begin in childhood and should leverage psychological interventions in order to prevent pain-related morbidities and promote better overall well-being in adulthood [13]. Thus, research on nonpharmacological pain management strategies is needed in general, particularly in this underserved population.

While the prevalence of pain is high in survivors of childhood cancer, no evidence-based interventions for pain are available for youth survivors of childhood cancer [14]. Most promising for this population may be cognitive behavioral therapies, as they can effectively target activity limitations, depression and anxiety symptoms, and sleep disturbances related to pain. Cognitive behavioral therapies are effective in reducing the burden of pain in youths with chronic noncancer pain conditions [15]; however, there are also significant barriers to participation in clinic-based cognitive behavioral therapy programs. For example, parents may have to take time off work and take their children out of school to visit a psychologist (the professionals most often trained and competent to deliver cognitive behavioral therapy), a psychologist may not be affordable, or there may not be a psychologist available in some areas.

In response to these limitations and barriers, internet-delivered cognitive behavioral therapy (CBT) can address these significant barriers and has demonstrated feasibility and efficacy in youths with chronic pain [16]. An 8-week web-based cognitive behavioral therapy program called web-based management of adolescent pain (WebMAP) has been rigorously developed and tested in children and adolescents with various chronic pain conditions, and is feasible and acceptable for this group, demonstrating clinically significant reductions on primary targets including activity limitations and pain intensity [17,18]. In addition, previous trials with WebMAP have shown economic benefits for both intervention and education groups, demonstrating a significant decrease in health care–related expenditures from the year before participating in the trial to the year after [19]. In other populations, WebMAP has demonstrated feasibility and acceptability in adolescents with sickle cell disease and their parents, with no indication that the program needed to be significantly tailored for those with sickle cell disease [20].

Treatment for chronic pain in youths is enhanced by the inclusion of parents, given the developmentally important role they play in their child’s life. For example, unique social factors exist related to parent and family influences on pediatric chronic pain and disability such as parental responses to child pain behavior, parenting style, family environment, and overall family communication and functioning [21]. Parent pain catastrophizing has also been shown to play an important role in their child’s pain interference [22,23]. Parent protectiveness is also related to important child-level outcomes (eg, child functional disability) in trials assessing family cognitive behavioral therapy for adolescents with chronic pain [24]. WebMAP includes a parent component in which parents complete modules in parallel with their child in order to target these factors. In WebMAP, parents are taught various operant strategies to respond to their child’s pain behaviors. In previous trials with WebMAP, parent distress, and parent protective behaviors predict child functional disability trajectories 6- and 12-months post treatment [25], and parent engagement in the program may also be predictive of treatment outcomes [26]. Thus, the inclusion of parents in psychological intervention trials for youths with chronic pain is recommended.

WebMAP has never been tested on a sample solely comprised of youths with a history of cancer. Thus, the primary objective of this study is to test the acceptability and feasibility of WebMAP for survivors of childhood cancer living with chronic pain and their parents, using a single-arm, pretest-posttest mixed methods design with semistructured interviews. Objective 1 will test the feasibility and acceptability of WebMAP for a sample of survivors with chronic pain and their parents. We hypothesize that the intervention will be feasible, that families will be engaged in the treatment and it will be safe. Objective 2 will assess the acceptability of WebMAP using qualitative interviews. Objective 3 will assess WebMAP’s effect on activity limitations, pain intensity, depression and anxiety symptoms, and sleep disturbances. We hypothesize that survivors of childhood cancer participating in WebMAP will report significantly reduced activity limitations, pain, anxiety and depressive symptoms, and sleep disturbances from before to after the intervention. Finally, objective 4 will assess WebMAP’s effect on parent pain catastrophizing and parental response to their child’s pain. We hypothesize that parents participating in WebMAP will report significantly reduced catastrophizing about their child’s pain, distress, and reduced protective parental responses to their child’s pain. This study protocol follows the Standard Protocol Items: Recommendations for Interventional Trials Checklist for recommended items to address in a clinical trial protocol [27].

## Methods

### Study Design

This study will use a single-arm, mixed methods, pre-post design. Survivors of childhood cancer with chronic pain will participate in an 8-week internet-delivered cognitive behavioral therapy intervention (WebMAP). Assessment questionnaires will be collected before the intervention (T0), immediately following the intervention (T1), and 3-months after intervention completion (T2). Individual interviews will also be conducted upon completion of the intervention.

### Participants

#### Sample Size

Based on an audit of sample sizes of combined feasibility and pilot trials in similar populations, median sample sizes ranged from 30 to 36 [28]. A sample of 34 will be sufficient to detect an effect size (Cohen *d*) of 0.4 with our preliminary outcomes with 80% power and α at .05. Therefore, we will aim to recruit a total sample of 38 to conservatively account for 10% attrition over the study duration.

#### Eligibility Criteria

Inclusion criteria for participation are (1) history of any cancer diagnosis; (2) current age 10-17 years; (3) >2 years posttreatment or completed treatment and >5 years post diagnosis; (4) pain present over prior 3 months that impairs at least one area of daily life and occurs >1 per month; and (5) computer access, internet, and literacy. Survivors wishing to participate without a parent will not be excluded but will still require parental consent.

#### Patient Engagement

Patient engagement is an important component of health research. One systematic review found that across studies, engaging patients as partners in research increases enrollment and retention rates, and improves dissemination of results to be more meaningful and digestible for patient audiences [29]. The Canadian Institutes of Health Research Strategy for Patient Oriented Research Patient Engagement Framework [30] is a roadmap on how to engage patients as partners in health research, with the goal of improving health outcomes and creating a more enhanced health care system. The framework provides guiding principles (inclusiveness, support, mutual respect, and cobuilding of ideas), tools, and resources for researchers to use throughout their projects. It highlights that patients should be included from the start of the research project and that they should collaborate with the researchers as equal members of the team.

In this study, 3 patient partners were involved in the study design and will be involved in recruitment, interpretation of results, and knowledge translation for this study. More specifically, the patient partners have been consulted on the study protocol where they provided feedback and have approved the final version to be submitted to the institutional ethics committee. We started a group on Slack (Slack Technologies, Salesforce Inc), a web-based communication platform that allows us to share communications and materials all in 1 place. Patient partners will help with the creation and dissemination of recruitment materials, using their own connections with advocacy groups and Canadian cancer advocacy groups. Similar to previous experiences, we will discuss findings with patient partners in order to seek their interpretation of the results and how to communicate them. This work will foster inclusiveness, support, mutual respect, and cobuilding of ideas [30].

### Procedures

#### Recruitment

**Figure 1:**
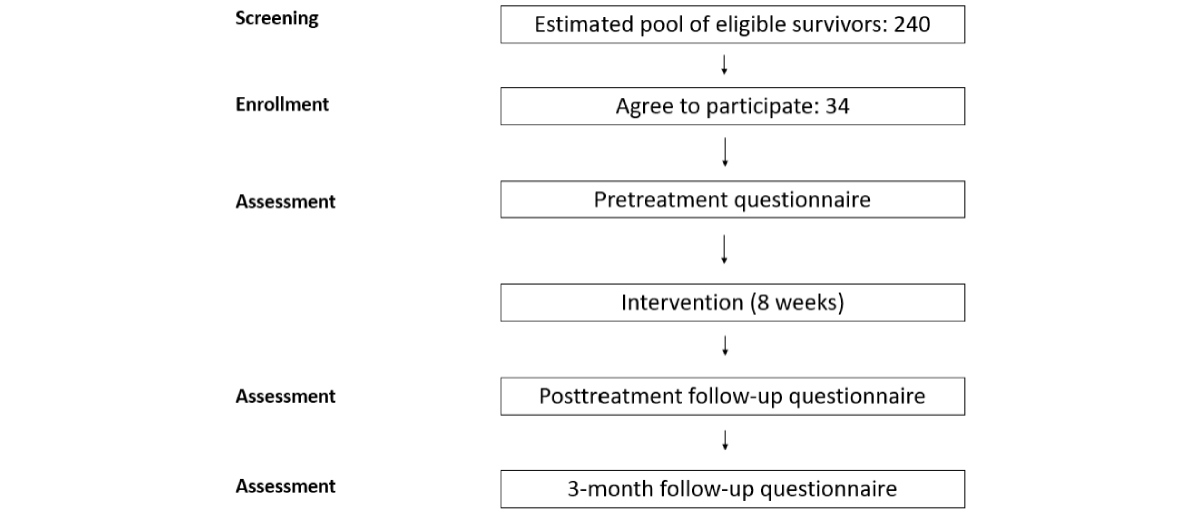
Participant recruitment flowchart.

Survivors will be recruited from the Long-Term Survivors Clinic at the Alberta Children’s Hospital in Calgary, Alberta, Canada. The participant flowchart is in Figure 1. The study team will contact potentially eligible survivors by phone or email. Approximately 700 survivors receive follow-up care at ACH, and an estimated 240 of these survivors may be experiencing chronic pain based on our research [3]. Approximately 94% of Canadians have internet access at home [31] and recruitment rates from our previous pain-related research were 70% [3]. Conservatively, we expect 40% of these survivors to meet our inclusion and exclusion criteria, leaving a possible sample of 70. Meeting our targets would require a participation rate of 49% (34/70).

#### Data Collection

Upon enrollment and completion of informed consent, survivors and their parents will complete pretreatment questionnaires (T0) and be given access to WebMAP. Upon treatment completion (T1), survivors and parents will complete the same questionnaire battery plus the Treatment Evaluation Inventory—Short Form [32]. In addition, we will conduct posttreatment interviews to gather feedback on the program. Patient and caregiver partners will be involved in creating the interview questions and conducting a portion of the interviews. Interviews will be recorded and transcribed. Finally, survivors will be administered the same questionnaire battery at 3 months post intervention (T2) to assess longer-term effects of the intervention. Questionnaires will be completed over internet via Research Electronic Data Capture (REDCap) tools hosted by the University of Calgary Clinical Research Unit. REDCap is a secure, web-based application designed to support data capture for research studies, providing (1) an intuitive interface for validated data entry, (2) audit trails for tracking data manipulation and export procedures, (3) automated export procedures for seamless data downloads to common statistical packages, and (4) procedures for importing data from external sources. The investigator leading the trial (MP) and the sponsor (FS) will have access to the final trial data set (Table 1).

**Table 1:** World Health Organization trial registration data set.

Data category			Information
Primary registry and trial identifying number			ClinicalTrials.gov NCT05241717
Date of registration in primary registry			February 4, 2022
Sources of monetary or material support			Alberta Children’s Hospital Trainee Small Research Grant^a^
Primary sponsor			Fiona Schulte, PhD^a^, fsmschul@ucalgary.ca
Contact for public queries			Fiona Schulte, PhD, fsmschul@ucalgary.ca
Public title			Internet-delivered cognitive behavioral treatment for adolescent survivors of childhood cancer with chronic pain
Scientific title			Internet-delivered cognitive behavioral treatment for adolescent survivors of childhood cancer with chronic pain: A pilot feasibility trial
Countries of recruitment			Canada
Health conditions or problems studied			Cancer, chronic pain
**Intervention**			
	Active comparator	Internet-delivered cognitive behavioral therapy	
**Key inclusion and exclusion criteria**			
	Ages eligible for study	10-17 years	
	Sexes eligible for study	Any	
	Accepts healthy volunteers	No	
	Inclusion criteria	History of any cancer diagnosis, >2 years post treatment or completed treatment and >5 years post diagnosis, pain present over prior 3 months that impairs at least one area of daily life and occurs >1 per month, computer access and literacy.	
Study type			Interventional study; intervention model: single arm; primary purpose: pain management; phase II
Date of first enrollment			May 24, 2022
Target sample size			34
Recruitment status			Currently recruiting
Primary outcomes			Feasibility, satisfaction
Key secondary outcomes			Pain intensity, activity limitations, anxiety symptoms, depressive symptoms, pain interference, insomnia symptoms, parent catastrophizing about child’s pain, parent distress, parent responses to child’s pain behaviors

^a^The funders will not contribute to any aspects of study design or management. The sponsor will be responsible for overseeing the study, interpretation of data, and the decision to submit the report for publication.

### Intervention

Upon completion of the pretreatment questionnaires, survivors will receive access to the WebMAP program, an 8-week web-based cognitive behavioral therapy program with weekly modules including (1) education about chronic pain, (2) recognizing stress and negative emotions, (3) deep breathing and relaxation, (4) implementing coping skills at school, (5) cognitive skills, (6) sleep hygiene and lifestyle, (7) staying active, and (8) relapse prevention.

WebMAP is built using a responsive framework so that it can be easily viewed on any internet-enabled device (eg, smartphone and laptop). The program makes use of multimedia components (eg, audio and video) and interactive exercises. Support is provided through a message center, which allows asynchronous messaging with a coach over the internet. The study coach will be a member of the research team (MP) and is a clinical psychology doctoral student trained in cognitive behavioral therapy. The study coach will have access to a previously developed web-based coach’s manual to standardize responses to participants in the message center. The WebMAP backend system monitors the completion of modules so treatment progress can be recorded.

Survivors will be asked to complete 1 module per week, which is approximately 20 minutes in length. Survivors and parents will be asked to spend approximately 4.5 hours each over the course of the intervention on WebMAP including 4 hours to complete the modules and 30 minutes total corresponding with a coach.

Each week, survivors will be instructed to log in to WebMAP to learn a new skill and practice that skill for 1 week to allow time for skills acquisition. WebMAP consists of 2 separate, password-protected programs including 1 for children and adolescents and 1 for parents.

Parents will also be given access to their own web-based program with weekly modules including (1) education about chronic pain, (2) recognizing stress and negative emotions, (3) operant strategies I (using attention and praise to increase positive coping), (4) operant strategies II (using reward to increase positive coping and strategies to support school goals), (5) modeling, (6) sleep hygiene and lifestyle, (7) communication, and (8) relapse prevention. The parent version of WebMAP uses the same type of multimedia and interactive elements as the teen version including access to the message center.

### Measures

#### Clinical and Demographic Information

We will collect clinical and demographic information from parents including survivor’s date of birth, gender, race, ethnicity, and household income. We will also ask about the survivor’s previous medical issues (excluding cancer) including major injuries, illnesses, or surgeries as well as current or past history of pain treatments (behavioral or pharmacological). Upon receiving consent, we will also collect clinical information from electronic medical records including survivor sex, diagnosis date and description, and treatment received.

#### Objective 1

##### Feasibility

Feasibility will be assessed by considering rates of: (1) recruitment, (2) retention, (3) treatment engagement, (4) acceptability, and satisfaction. “Recruitment” will be measured by the number of survivors enrolled or consented divided by the total number of survivors invited to participate. Based on previous trials of similar nature [17], a 50% recruitment rate of eligible survivors will be used as the benchmark to determine this factor of study feasibility. “Retention” will be measured by the number of survivors who complete the study defined as those who complete the intervention and all assessments from enrollment (T0) to 3-month follow-up (T2) divided by the total number of survivors who enrolled in the study. Survivors will be considered to be enrolled in the study when they have read and signed the informed consent form. Based on previous pilot trials [17], an 80% retention rate will be considered “feasible.” “Treatment engagement” will be measured by number of modules completed by survivors and parents. Based on similar trials [17], the goal to determine treatment engagement will be at least 75% of survivors completed at least 6 of the 8 modules, and the mean number of modules completed by the sample being at least 6. We hypothesize that >75% of survivors will complete assessment measures across the 3 specified time points. “Acceptability” will be measured using the Treatment Evaluation Inventory—Short Form (TEI-SF) [32], a 9-item self-report measure of acceptability of treatment, completed by both survivors and parents. The TEI-SF was originally designed to measure parents’ acceptability of a treatment for children with problem behaviors but has been adapted to assess the acceptability of WebMAP in previous studies [17]. Responses are scored on a 5-point Likert scale and summed to create a total score. Higher scores represent better acceptability. Moderate to high acceptability, defined as a score of 27 or higher, has been considered sufficient for trials of similar nature [17,33]. The TEI-SF has good reliability and validity across various child treatments [32]. This study will use the adapted version and will be completed by survivors and parents.

##### Satisfaction

A survey including open-ended questions will be created with patients to elicit feedback on specific modules. These questions will ask about what information was helpful and what may need to be adjusted to better serve youths with a history of cancer for both patients and parents. The satisfaction survey will be completed as part of the posttreatment questionnaire by survivors and parents.

##### Qualitative Interviews

Semistructured interviews will be conducted with survivors and parents separately upon treatment completion to assess feasibility, acceptability, and satisfaction with the program. Patient partners will be involved in creating an interview guide. The interview guide will include open-ended questions about participants’ experience with the program to facilitate self-reflection and open discussion around important aspects of their experience. Interviews will be audio-recorded and transcribed verbatim.

#### Objective 2

##### Pain Intensity

Daily surveys, administered to survivors through REDCap, will be used to assess the daily presence of pain and pain intensity for 7 days at the beginning of each assessment period (T0, T1, and T2). Pain intensity will be assessed with an item asking about the magnitude of pain experienced answered on an 11-point numerical rating scale (0=no pain, 10=worst pain). This measure of pain intensity has demonstrated to be a reliable and valid measure of pain in many populations of children 8 years and older [34-37], including those with chronic cancer pain [38].

##### Activity Limitations

The Child Activity Limitations Interview (CALI-9) [39] is a measure of children’s perceived difficulty in completing typical daily activities because of pain. The CALI will be collected from survivors for 7 days at the beginning of each of the study timepoints (pretreatment, posttreatment, and 3-month follow-up). Survivors will choose from a list of 9 activities (eg, running, doing a hobby, playing with friends, sports, going to school), and will be asked each day whether the activity occurred and how difficult the activity was to perform because of their pain. Total scores range from zero to 100, where higher scores indicate greater functional limitations. Average daily limitation scores across each assessment period will be computed. The CALI has demonstrated reliability and validity in school-age children and adolescents with chronic pain, including those referred to pain clinics from hematology departments [40].

##### Anxiety and Depressive Symptoms and Pain Interference

The Patient-Reported Outcomes Measurement Information System (PROMIS) Anxiety and Depression instruments [41] (8-item short form) will be administered to survivors to screen for current symptoms of anxiety and depression. Survivors will complete the self-report instrument corresponding to their age (child: aged 8-17 years; adult: aged 18 years or older). Items (eg, “I felt worried”) are rated on a 5-point scale (1=never, 5=always). Higher scores signify greater severity of symptoms. Pain interference will be assessed using the 4-item PROMIS interference scale for survivors 8-17 years of age, and the 7-item PROMIS interference scale for survivors over 18 years of age [42]. The PROMIS pediatric measures have been validated for use in pediatric oncology [42].

##### Insomnia

Insomnia Severity Index (ISI) is a 7-item self-report questionnaire assessing the nature, severity, and impact of insomnia [43,44] and will be administered to survivors. Items are rated on a 5-point Likert scale (0=no problem; 4=very severe problem). The scale is scored using a total sum ranging from 0 to 28 and cut scores are interpreted as such: absence of insomnia (0-7), subthreshold insomnia (8-14), moderate insomnia (15-21), and severe insomnia (22-28). The ISI is a reliable and valid measure used to detect cases of insomnia in a population sample and is sensitive to treatment response in patients presenting with sleep difficulties [43].

##### Catastrophizing About Child’s Pain

The Pain Catastrophizing Scale–Parent Version [45] is a 13-item measure used to assess parents’ catastrophic thoughts and feelings about their child’s pain and will be administered over the internet via REDCap. Parents rate statements about their child’s pain on a 5-point Likert scale (ie, “When my child has pain, I can’t keep it out of my mind”). Items are summed to yield a total score and can also be summed to provide scores on 3 subscales: rumination, magnification, and helplessness. Lower scores indicate less catastrophizing. This scale has been found to have good reliability in parents with children who experience pain.

##### Parental Responses to Child’s Pain Behaviors

The Adult Responses to Children’s Symptoms [46,47] measures parental responses to their child’s pain behaviors and will be administered over the internet via REDCap. This study will only use the Protect subscale, which assesses parents’ responses that either positively or negatively reinforce pain complaints. Parents’ responses are rated on a 5-point scale (0=never, 4=always). Subscale scores represent an average of the items, with higher scores indicating more frequent use of that behavioral style. This scale has demonstrated reliability and validity for use with parents of children with chronic pain [46,47].

##### Parent Distress

The Kessler Psychological Distress Scale [48] will be administered to parents and is a 6-item measure used to assess nonspecific psychological distress representing diagnoses of major depression and generalized anxiety disorder and also contains a positive affect domain. The measure has good precision in the 90th-99th percentile range of the population, and has solid psychometric properties across major sociodemographic populations distribution in the United States and Canada [48]. It also strongly discriminates between meeting and not meeting diagnostic thresholds for psychological disorders [48].

### Data Analysis

#### Quantitative Data Analysis

To address objective 1, we will use frequencies and descriptive statistics to describe recruitment and retention rates, treatment adherence, and acceptability. To address objectives 2 and 3, primary efficacy end points of the study will be calculated as within-person changes in outcome measures using Cohen *d* effect sizes from T0 to T1 and T2. Outcome data will be analyzed using paired samples *t* tests in order to assess changes in outcome variables over time. Treatment effect sizes (Cohen *d*) will be calculated for changes in means, standardized by the pooled SD. All quantitative analyses will be conducted using SPSS (version 27; IBM Corp).

#### Qualitative Data Analysis

Qualitative data will be coded independently by 2 study team members and analyzed using reflexive thematic content analysis, consistent with Braun and Clark’s thematic analysis guide [49]. Team members will first familiarize themselves with the data by reading the transcripts multiple times. The data will then be coded independently using a hybrid deductive-inductive approach whereby codes will be inductively derived from the data and codes will be deductively generated from existing literature [50]. Afterward, the 2 coders and the principal investigator (MP) will meet to discuss the codes and iteratively generate themes and subthemes. Additional team members will be invited to offer critical reflections and insight on the interpretations. Several strategies will be used throughout the data analysis to enhance rigor including having clear and extensive descriptions around the research setting and process, member checking, providing thick descriptions of themes, regular debriefing meetings, data triangulation with the quantitative analysis, and having the authors actively practicing reflexivity through notes and meetings [51].

### Ethics Approval

Ethics approval was obtained from the Health Research Ethics Board of Alberta Cancer Committee (HREBA.CC-21-0272). Any changes to this protocol will be made through this ethics board. Potential survivors will be contacted and informed of study details such as the voluntary nature of participation, confidentiality, and risks and benefits of participating. Once survivors are fully informed, they will be given the opportunity to provide written consent and enroll in the study. Survivors will provide assent and will require parental consent to participate. Parents will provide consent for their own participation. WebMAP is known to be safe and previous trials reported no adverse events directly related to study participation [17]. Data will be collected using participant identification numbers to protect confidentiality. Names and corresponding identification numbers will be kept in a separate, password-protected document. Potential benefits of participating in the intervention include a reduction in pain and an improvement in mental health.

The results will be disseminated to a range of stakeholders such as survivors of childhood cancer, parents of survivors of childhood cancer, health care practitioners, and researchers. Dissemination efforts will be made through national and international conference presentations, social media posts (eg, Twitter and Facebook), peer-reviewed academic journal publications, and cancer advocacy group newsletters (eg, Kids Cancer Care).

## Results

This study was funded in January 2022. Data collection started in June 2022 and is projected to finish by August 2023. As of December 2022, we have recruited 10 participants, with zero participants completing all aspects of the protocol.

## Discussion

The primary objective of this study is to assess the feasibility and acceptability of a well-studied internet-based psychological intervention developed for adolescents with chronic pain in a population of youths with a history of cancer. The strength of the study is the usage of a mixed methods design, which will provide both quantitative and qualitative information regarding the feasibility, acceptability, and potential efficacy of the intervention. Additionally, 3 patient and caregiver partners with lived experience of cancer and chronic pain will inform the study from the beginning. The web-based intervention will also be accessible for survivors who would not otherwise be able to access CBT with a psychologist. This may prove to be more inclusive for survivors living in rural or remote areas and survivors of families with low household incomes.

The proposed study has limitations. Participants for previous studies were recruited from tertiary pain clinics, which serve the most severe cases of chronic pain. One limitation of this study is that the participants may not experience a severe chronic pain problem compared to participants from other studies, though screening criteria require that they have chronic pain that affects at least one area of their daily life, similar to previous studies. This limitation may lead to smaller effects of the pain intervention on participants with mild to moderate chronic pain problems, and may also affect motivation to engage in the program if participants are generally functioning with pain. Another limitation is that the study will be conducted at a single location, in which the feasibility and acceptability of the study may not be generalizable to survivors across Canada.

The Obesity-Related Behavioral Intervention Trials (ORBIT) Model for Behavioral Treatment Development [52] is a framework for increasing the number of optimized evidence-based behavioral trials to prevent and treat chronic diseases. The ORBIT model includes several phases that serve as a roadmap for conducting behavioral intervention research in a systematic way that ensures quality and cost-efficient methods. This dynamic framework begins with a significant clinical question. This study proposes that while chronic pain is a prevalent issue in survivors of childhood cancer, no interventions to date have been empirically tested on this population. Phase I in the ORBIT Model represents the Design phase, where the goal is to design essential features of the intervention or to adapt to an existing treatment [52]. This phase has already been completed in that the intervention has been designed, refined, and tested on a different population [17]. Phase II represents the Preliminary Testing phase, where the goal is to test the ability of a fixed intervention to produce a clinically significant improvement on a behavioral risk factor [52]. While this phase has already been completed in previous studies [17], this intervention has never been tested on adolescents with a history of cancer, and thus this study aims to complete this phase. Should survivors not find the study helpful and adjustments are warranted, the trial will move back to phase I to be tailored for youths with a history of cancer. If the study finds WebMAP to be feasible and acceptable with promising preliminary efficacy, the trial will move to a phase III efficacy trial in future studies. This study will provide valuable information about the potential usefulness of WebMAP for survivors of childhood cancer with chronic pain.

## Abbreviations

CALI: Child Activity Limitations Interview
CBT: cognitive behavioral therapy
ISI: Insomnia Severity Index
ORBIT: Obesity-Related Behavioral Intervention Trials
PROMIS: Patient-Reported Outcomes Measurement Information System
REDCap: Research Electronic Data Capture
TEI-SF: Treatment Evaluation Inventory—Short Form
WebMAP: web-based management of adolescent pain

## Data Availability

Data and materials will be available upon request of the corresponding author after data have been collected.
